# Effects of Selen on the Antidepressant-like Activity of Agents Affecting the Adenosinergic Neurotransmission

**DOI:** 10.3390/metabo12070586

**Published:** 2022-06-23

**Authors:** Aleksandra Szopa, Mariola Herbet, Ewa Poleszak, Karolina Bogatko, Marta Ostrowska-Leśko, Katarzyna Świąder, Jarosław Szponar, Anna Serefko

**Affiliations:** 1 Laboratory of Preclinical Testing, Chair and Department of Applied and Social Pharmacy, Medical University of Lublin, 1 Chodźki Street, 20-093 Lublin, Poland; ewa.poleszak@umlub.pl (E.P.); karolina.bogatko@umlub.pl (K.B.); 2 Chair and Department of Toxicology, Medical University of Lublin, 8 Chodźki Street, 20-093 Lublin, Poland; mariola.herbet@umlub.pl (M.H.); marta.ostrowska-lesko@umlub.pl (M.O.-L.); 3 Chair and Department of Applied and Social Pharmacy, Medical University of Lublin, 1 Chodźki Street, 20-093 Lublin, Poland; katarzyna.swiader@umlub.pl; 4 Clinical Department of Toxicology and Cardiology, Stefan Wyszyński Regional Specialist Hospital in Lublin, Al. Kraśnicka 100, 20-550 Lublin, Poland; jaroslawszponar@umlub.pl; 5 Toxicology Clinic, Medical University of Lublin, Al. Kraśnicka 100, 20-550 Lublin, Poland

**Keywords:** selective antagonist of adenosine receptors, DPCPX, istradefylline, sodium selenite, forced swim test, tail suspension test, BDNF, gene expression, mice

## Abstract

The main goal of this study was to determine the antidepressant-like potential of the co-administration of sodium selenite (Se) and the selective adenosine A1 and A2A antagonists DPCPX and istradefylline (IST), respectively, in mice despair tests. Biochemical studies were performed to elucidate the action mechanisms of the investigated treatment strategies. The results confirmed that, when administered by itself, Se exerts an antidepressant-like effect in the FST and TST and that this activity is dose-dependent. Further experiments demonstrated that Se (0.25 mg/kg) significantly enhanced the activity of mice in both tests when co-administered with DPCPX (1 mg/kg) and IST (0.5 mg/kg) at doses which would be ineffective if administered individually. Our research revealed that neither DPCPX, IST, nor Se or combinations of the tested substances induced significant changes in the brain-derived neurotrophic factor (BDNF) levels in mice serum vs. the NaCl-treated group. However, we observed a decrease in the mRNA level of antioxidant defense enzymes. Molecular studies also showed changes in the expression of the *Slc6a15*, *Comt*, and *Adora1* genes, particularly after exposure to the combination of Se and DPCPX, which indicates a beneficial effect and may help to explain the key mechanism of the antidepressant effect. The combination of Se with substances attenuating adenosine neurotransmission may become a new therapeutic strategy for patients with depression.

## 1. Introduction

Selenium, a mineral substance present in trace amounts in the human body, has a number of biological functions [1,2]. It is essential for the proper functioning of selenoproteins, which are involved, inter alia, in oxidative stress defense and the metabolism of thyroid hormones [3,4]. One of the components of selenoproteins, selenocysteine, is located in the active center of many enzymes including, among others, in iodothyronine deiodinase, glutathione peroxidase, methionine, and thioredoxin reductase [4,5], and it is indispensable to the proper course of reactions mediated by these enzymes. Selenium, acting as an antioxidant [6], protects brain structures from damage by free radicals in the central nervous system (CNS). This effect is attributed primarily to selenoenzymes (mainly glutathione peroxidase and thioredoxin reductase, mentioned above) [7], but selenium can also have a direct impact on the CNS if used as selenate [7,8,9,10]. Likewise, the neuroprotective effect of selenium is also attributed to its impact on the influx of calcium ions into the cell [11,12] and anti-inflammatory properties [13,14,15,16]. Furthermore, selenium modulates various neurotransmitter systems, including the serotonergic [17], noradrenergic [18], dopaminergic [19], and glutamatergic [20] systems, which are involved in the physiopathology of mental disorders.

Therefore, selenium is considered to be crucial for the functioning of the brain and the CNS. In recent years, interest in the role of this trace element in the pathophysiology of CNS diseases, including depression, has increased due to the neuromodulatory effect of selenium on brain functions [21,22,23]. Both preclinical and clinical studies indicated an association between decreased selenium levels and the risk of developing depression. Mitchell et al. [24] found that selenium deficiency in rats is correlated with decreased levels of the brain-derived neurotrophic factor (BDNF), which has been extensively associated with the pathophysiology of major depressive disorder [25]. Several preclinical studies demonstrated that supplementation with both organic (e.g., m-trifluoromethyl-diphenyl diselenide, methyl phenyl selenide, and 3-(4-fluorophenylselenyl)-2,5-diphenylselenophene) and inorganic (sodium selenite) forms of selenium results in antidepressant- and anxiolytic-like effects in mice [26,27,28,29]. Clinical studies carried out in recent years also confirmed the relationship between selenium intake and the likelihood of developing major depressive disorder or anxiety behavior (for review see: [9,30,31]).

According to the literature, currently used therapeutic methods in patients suffering from depression (both pharmacotherapy, psychotherapy, and alternative therapies, e.g., phototherapy, phytotherapy, and acupuncture, etc.) are not sufficiently effective. It is estimated that commonly applied antidepressants are efficacious only in 50–75% of depressed people, and, moreover, their usage carries a number of side effects (including neurological, sexual, cardiovascular, anticholinergic, and gastrointestinal effects, etc.), which are the main cause of non-compliance and the discontinuation of pharmacotherapy [32,33,34,35]. Additionally, the desired therapeutic effect does not manifest itself after the administration of a single dose. Typically, concerning these drugs, antidepressant activity occurs after at least 4–6 weeks of regular use [35]. Therefore, new strategies for depression therapy are being sought. Recently, researchers have been focusing more and more on agents with mechanisms of action that differ from those of commonly prescribed antidepressants or on the possibility of using combinations of two or more substances with potential antidepressant-like effects. Such approaches aim to achieve safer and more effective therapy as well as faster improvement in patients with depressive disorders.

Recently, a substantial amount of data have highlighted the role of the adenosine system in the pathophysiology and therapy of depression and a relationship between selenium concentration and depressive disorders. Therefore, the main goal of the present study was to determine the antidepressant-like potential of the co-administration of the inorganic salt of selenium, sodium selenite (Se), with selective adenosine A1 and A2A antagonists, DPCPX and istradefylline (IST), respectively, in mice despair tests (i.e., in the forced swim test—FST and in the tail suspension test—TST). Biochemical and molecular studies were performed to elucidate the mechanisms of action of the investigated treatment strategies. The serum level of BDNF along with the expression of selected antioxidant defense genes (*Ogg1*, *MsrA*, and *Nrf2*) and genes responsible for changes in transduction and neuronal excitability in neuronal cells (*Slc6a15*, *Comt*, *Adora1*) were determined.

## 2. Results

### 2.1. Behavioral Tests

#### 2.1.1. Forced Swim Test (FST) and Tail Suspension Test (TST)

**Figure 1:**
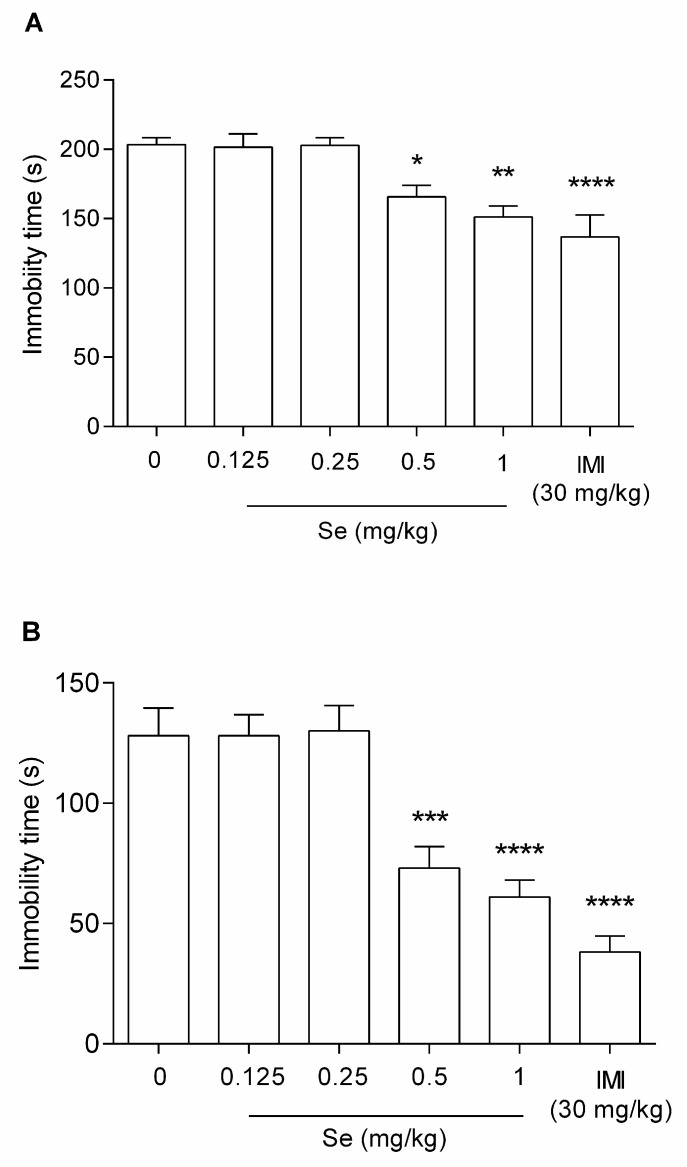
The antidepressant-like activity of sodium selenite in the FST (**A**) and the TST (**B**) in mice. Sodium selenite (Se) and NaCl 0.9% were administered i.p. 30 min, while IMI was given 60 min prior behavioral testing. Data are presented as the means ± SEM. Each experimental group consisted of 8–10 mice. * *p* < 0.05, ** *p* < 0.01, *** *p* < 0.001, **** *p* < 0.0001 vs. control group (one-way ANOVA followed by Dunnett’s post-hoc test).

*Sodium selenite dose-effect relationship.* To determine the antidepressant-like effect of sodium selenite (Se) and to select a suitable sub-therapeutic dose for further study, Se was administered at the following doses: 0.125, 0.25, 0.5, and 1 mg/kg. IMI was used as a reference drug at 30 mg/kg (Figure 1A—FST and 1B—TST) [one-way ANOVA: F(5,49) = 9.193; *p* < 0.0001; F(5,52) = 20.41; *p* < 0.0001, respectively]. 

Statistical analysis of the FST outcomes (Figure 1A) showed that Se at doses of 0.5 and 1 mg/kg significantly increased mice motility (* *p* < 0.05 and ** *p* < 0.01, respectively), whereas doses of 0.125 and 0.25 mg/kg did not have a significant influence on animals’ behavior (*p* > 0.05). Similarly, statistical analysis of the TST results demonstrated that Se at doses of 0.5 and 1 mg/kg significantly increased mice motility (*** *p* < 0.001 and **** *p* < 0.0001, respectively), whereas doses of 0.125 and 0.25 mg/kg did not have a significant influence on animals’ behavior (*p* > 0.05).

The dose of 0.25 mg/kg of Se was selected as a sub-therapeutic dose based on the carried-out analysis, and it was used in further experiments.

**Figure 2:**
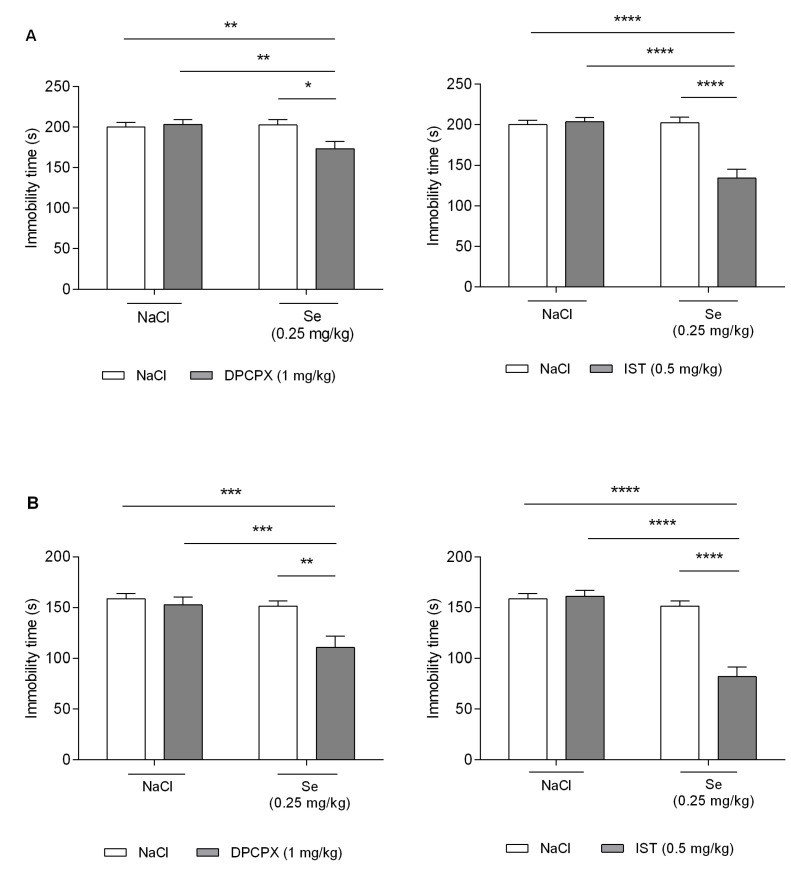
Effect of combined administration of sodium selenite and selective adenosine receptor antagonists in the FST (**A**) and the TST (**B**) in mice. DPCPX, sodium selenite (Se), and NaCl 0.9% were administered i.p. 30 min, while IST was administered p.o. 60 min prior to behavioral testing. Data are presented as the means ± SEM. Each experimental group consisted of 8–10 mice. * *p* < 0.05, ** *p* < 0.01, *** *p* < 0.001, **** *p* < 0.0001 vs. respective group (two-way ANOVA followed by Bonferroni’s post-hoc test).

*Influence of selective adenosine A1 and A2A receptor antagonists on the antidepressant-like activity of sodium selenite*. As showed in Figure 2, neither DPCPX nor IST or Se administered at sub-therapeutic doses (1 mg/kg, 0.5 mg/kg, and 0.25 mg/kg, respectively) considerably changed the immobility time of mice in both the FST and the TST (*p* > 0.05). In turn, the co-administration of DPCPX (1 mg/kg) and IST (0.5 mg/kg) with Se (0.25 mg/kg) at sub-therapeutic doses caused a significant decrease in the immobility time of mice when compared to the respective control group in both carried-out behavioral tests (Figure 2A—FST, 2B—TST).

In the FST (Figure 2A), two-way ANOVA indicated a significant DPCPX–Se interaction [F(1,34) = 5.75; *p* = 0.0222], with no significant effect in terms of both DPCPX [F(1,34) = 3.80; *p* = 0.0594] and Se [F(1,34) = 4.10; *p* = 0.0508], and a significant IST–Se interaction [F(1,36) = 22.86; *p* < 0.0001], with a significant effect in terms of both IST [F(1,36) = 18.64; *p* = 0.0001] and Se [F(1,36) = 19.80; *p* < 0.0001].

In the TST (Figure 2B), two-way ANOVA indicated a significant DPCPX–Se interaction [F(1,36) = 4.99; *p* = 0.0318], with a significant effect in terms of both DPCPX [F(1,36) = 9.29; *p* = 0.0043] and Se [F(1,36) = 10.18; *p* = 0.0029], and a significant IST–Se interaction [F(1,36) = 29.10; *p* < 0.0001], with a significant effect in terms of both IST [F(1,36) = 25.64; *p* < 0.0001] and Se [F(1,36) = 42.38; *p* < 0.0001].

#### 2.1.2. Spontaneous Locomotor Activity Test

As shown in Table 1, none of the examined doses of Se (0.125, 0.25, 0.5, and 1 mg/kg) (one-way ANOVA: [F(4,45) = 1.397; *p* = 0.2504]) affected the spontaneous locomotor activity of animals. Also, none of the tested compounds alone or in combination (Table 2) significantly changed the spontaneous locomotor activity of mice. In the case of the co-administration of DPCPX (1 mg/kg) and IST (0.5 mg/kg) with Se (0.25 mg/kg), two-way ANOVA indicated no significant DPCPX-Se and IST-Se interaction ([F(1,36) = 0.14; *p* = 0.7109] and [F(1,36) = 2.06; *p* = 0.1602], respectively).

### 2.2. Evaluation of the BDNF Level

**Figure 3:**
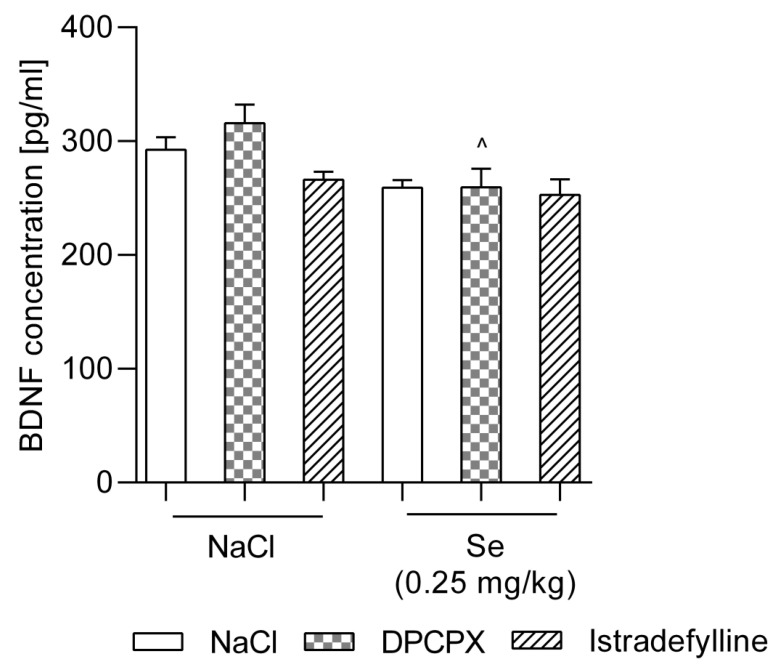
Effect of combined administration of sodium selenite, and selective adenosine receptor antagonists on the BDNF level in murine serum. DPCPX, sodium selenite (Se), and NaCl 0.9% were administered i.p. 30 min, while IST was administered p.o. 60 min prior behavioral testing. Data are presented as the means ± SEM. Each experimental group consisted of 8–10 mice. ^ *p* < 0.01 vs. DPCPX (two-way ANOVA followed by Bonferroni’s *post hoc* test).

As shown in Figure 3, neither DPCPX (1 mg/kg) nor IST (0.5 mg/kg) or Se (0.25 mg/kg) administered alone induced statistically significant changes in the BDNF level in mice serum (*p* > 0.05) compared to the NaCl-treated group. Only in the group which received DPCPX + Se was a significant decrease in the level of BDNF noticed when compared to the DPCPX-treated group (*p* < 0.05). There were no statistically significant changes compared to the NaCl-treated group in any of the groups which received the combination of tested compounds (*p* > 0.05).

### 2.3. Evaluation of the Relative mRNA Level of Selected Gens

**Figure 4:**
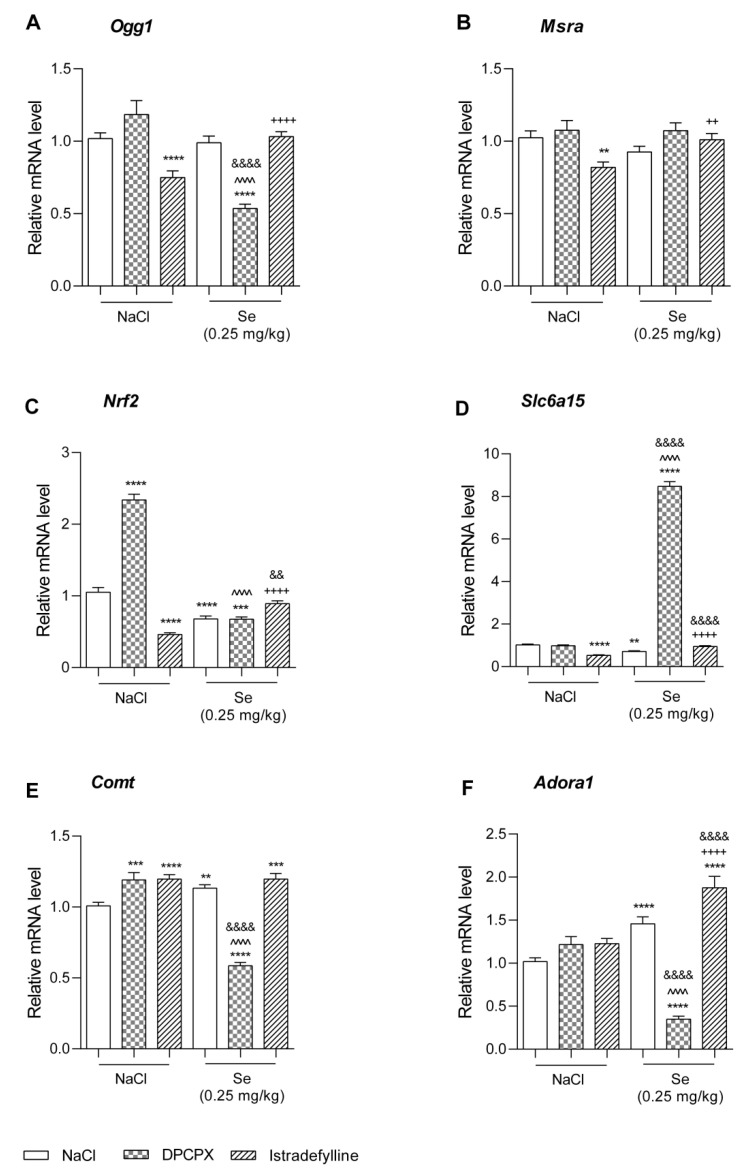
Effect of combined administration of sodium selenite and selective adenosine receptor antagonists on the expression of gens in murine prefrontal cortex: (**A**) *Ogg1*, (**B**) *Msra*, (**C**) *Nrf2*, (**D**) *Slc6a15*, (**E**) *Comt*, (**F**) *Adora1*. DPCPX, sodium selenite (Se), and NaCl 0.9% were administered i.p. 30 min, while IST was administered p.o. 60 min prior behavioral testing. Data are presented as the means ± SEM. Each experimental group consisted of 8–10 mice. ** *p* < 0.01, *** *p* < 0.001, **** *p* < 0.0001 vs. NaCl; ^^^^ *p* < 0.0001 vs. DPCPX; ^++^ *p* < 0.01, ^++++^ *p* < 0.0001 vs. IST; ^&&^ *p* < 0.01, ^&&&&^ *p* < 0.0001 vs. Se (two-way ANOVA followed by Bonferroni’s post hoc test).

The effect of DPCPX (1 mg/kg), IST (0.5 mg/kg), Se (0.25 mg/kg), and the co-administration of DPCPX (1 mg/kg) or IST (0.5 mg/kg) with Se (0.25 mg/kg) on the expression of selected gens in murine prefrontal cortex is illustrated in Figure 4.

*Ogg1*. As presented in Figure 4A, the administration of IST alone and the co-administration of DPCPX + Se significantly decreased the relative mRNA level of *Ogg1* when compared to the NaCl-treated group (*p* < 0.0001). The co-administration of DPCPX with Se resulted in a significant decrease in the *Ogg1* expression in comparison to both the DPCPX- and Se-treated groups (*p* < 0.0001). In turn, in animals that received IST + Se, a statistically significant increase in the *Ogg1* mRNA level was observed vs. the IST-treated group (*p* < 0.0001).

A two-way ANOVA indicated a significant DPCPX–Se interaction [F(1,68) = 30.86; *p* < 0.0001], with a significant effect in terms of both DPCPX [F(1,68) = 6.56; *p* = 0.0127] and Se [F(1,68) = 36.75; *p* < 0.0001], and a significant IST–Se interaction [F(1,91) = 13.23; *p* = 0.0005], with a significant effect in terms of both IST [F(1,91) = 7.18; *p* = 0.0088] and Se [F(1,91) = 8.82; *p* = 0.0038].

*Msra.* As presented in Figure 4B, the administration of IST alone significantly decreased the relative mRNA level of *Msra* when compared to the NaCl-treated group (*p* < 0.01). The co-administration of IST with Se resulted in a significant increase in the *Msra* expression in comparison to the IST-treated group (*p* < 0.01).

A two-way ANOVA indicated no significant DPCPX–Se interaction [F(1,68) = 0.80; *p* = 0.3729], with no significant effect in terms of both DPCPX [F(1,68) = 3.42; *p* = 0.0686] and Se [F(1,68) = 0.90; *p* = 0.3463], and a significant IST–Se interaction [F(1,92) = 11.89; *p* = 0.0009], with no significant effect in terms of both IST [F(1,92) = 2.03; *p* = 0.1576] and Se [F(1,92) = 1.19; *p* = 0.2776].

*Nrf2*. As presented in Figure 4C, the administration of DPCPX alone significantly increased, whereas treatment with IST and Se decreased, the relative mRNA level of *Nrf2* when compared to the NaCl-treated group (*p* < 0.0001). The co-administration of DPCPX with Se resulted in a significant decrease in *Nrf2* expression in comparison to the NaCl- and DPCPX-treated groups (*p* < 0.001 and *p* < 0.0001, respectively). Additionally, in the case of the IST + Se group, a significant increase in the *Nrf2* mRNA level vs. the IST- and Se-treated groups was noted (*p* < 0.0001 and *p* < 0.01, respectively).

A two-way ANOVA indicated a significant DPCPX–Se interaction [F(1,68) = 106.50; *p* < 0.0001], with a significant effect in terms of both DPCPX [F(1,68) = 104.45; *p* < 0.0001] and Se [F(1,68) = 263.87; *p* < 0.0001], and a significant IST–Se interaction [F(1,92) = 78.01; *p* < 0.0001], with a significant effect in terms of IST [F(1,92) = 17.28; *p* < 0.0001] and no significant effect in terms of Se [F(1,92) = 0.43; *p* = 0.5140].

*Slc6a15*. As presented in Figure 4D, the administration of DPCPX and Se alone significantly decreased the relative mRNA level of *Slc6a15* when compared to the NaCl-treated group (*p* < 0.0001 and *p* < 0.01, respectively). The co-administration of DPCPX with Se resulted in a significant increase in *Slc6a15* expression in comparison to the NaCl-, DPCPX- and Se-treated groups (*p* < 0.0001). Additionally, in a case of the IST + Se group, a significant increase in the *Slc6a15* mRNA level vs. the IST- and Se-treated group was noted (*p* < 0.0001).

A two-way ANOVA indicated a significant DPCPX–Se interaction [F(1,68) = 2022.50; *p* < 0.0001], with a significant effect in terms of both DPCPX [F(1,68) = 1981.52; *p* < 0.0001] and Se [F(1,68) = 1717.57; *p* < 0.0001], and a significant IST–Se interaction [F(1,92) = 96.73; *p* < 0.0001], with a significant effect in terms of IST [F(1,92) = 12.74; *p* = 0.0006] and no significant effect in terms of Se [F(1,92) = 2.43; *p* = 0.1223].

*Comt.* As presented in Figure 4E, the administration of DPCPX, IST, and Se alone and the co-administration of IST with Se significantly increased the relative mRNA level of *Comt* when compared to the NaCl-treated group (*p* < 0.001, *p* < 0.0001, *p* < 0.01, and *p* < 0.001, respectively). The co-administration of DPCPX and Se resulted in a significant decrease in *Comt* expression in comparison to the NaCl-, DPCPX-, and Se-treated group (*p* < 0.0001).

A two-way ANOVA indicated a significant DPCPX–Se interaction [F(1,68) = 130.32; *p* < 0.0001], with a significant effect in terms of both DPCPX [F(1,68) = 32.36; *p* < 0.0001] and Se [F(1,68) = 56.16; *p* < 0.0001], and a significant IST–Se interaction [F(1,92) = 4.19; *p* = 0.0436], with a significant effect in terms of IST [F(1,92) = 4.18; *p* = 0.0437] and a significant effect in terms of Se [F(1,92) = 17.04; *p* < 0.0001].

*Adora1*. As presented in Figure 4F, only Se administered alone significantly increased the relative mRNA level of *Adora1* when compared to the NaCl-treated group (*p* < 0.0001). The co-administration of DPCPX with Se resulted in a significant decrease in *Adora1* expression in comparison to the NaCl-, DPCPX-, and Se-treated group (*p* < 0.0001). In turn, in a case of IST + Se group, a significant increase in the *Adora1* mRNA level vs. the NaCl-, IST- and Se-treated group was noted (*p* < 0.0001).

A two-way ANOVA indicated a significant DPCPX–Se interaction [F(1,56) = 64.86; *p* < 0.0001], with a significant effect in terms of both DPCPX [F(1,56) = 30.28; *p* < 0.0001] and Se [F(1,56) = 6.54; *p* = 0.0133], and a significant IST–Se interaction [F(1,64) = 74.57; *p* < 0.0001], with a significant effect in terms of IST [F(1,64) = 36.04; *p* < 0.0001] and a significant effect in terms of Se [F(1,64) = 7.98; *p* = 0.0063].

## 3. Discussion

*Behavioral studies.* As far as we know, this is the first study to evaluate the concomitant effect of DPCPX (a selective antagonist of adenosine A1 receptor) and istradefylline—IST (a selective antagonist of the adenosine A2A receptor) with a selenium compound (sodium selenite, Se) in two behavioral tests assessing their antidepressant potential, i.e., in the FST and in the TST. Both tests, referred to as despair behavioral tests, are used worldwide in pre-clinical laboratories [36,37].

At the first stage of our experiment, we confirmed that, Se when given at a single dose, exerts an antidepressant-like effect in the FST and in the TST and that this activity is dose-dependent. Mice that received either 0.5 or 1 mg/kg of Se as an intraperitoneal injection were highly active in both despair behavioral tests and their performance was similar to those observed after impramine (30 mg/kg, i.p.), a well-known antidepressant drug that served as a positive control in our study. However, lower tested doses of Se (i.e., 0.25 and 0.125 mg/kg) did not influence animals’ behavior in the applied tests. Our finding are in line with reports by other authors [31,38], who also demonstrated the antidepressant-like potential of selenium preparations. According to the meta-analysis by Sadat Sajjadi and colleagues [39], supplementation with selenium can be effective in reducing depressive symptoms, whereas a high selenium intake may have a protective role against postpartum depression. In contrast, Pasco et al. [23] suggested that selenium deficiency may be associated with the development of depression.

During the next step of our study, we investigated whether Se was able to interact with the A1 and A2A receptor antagonists, i.e., DPCPX and IST, respectively. We chose the above-mentioned selective ligands of adenosine receptors since in our previous project we had demonstrated their antidepressant-like potential [40,41,42,43]. Moreover, we found out that the administration of DPCPX may augment the activity of commonly used antidepressants (i.e., imipramine, escitalopram, reboxetine, moclobemide, venlafaxine, and bupropion) [42,43], whereas both DPCPX and IST are able to potentiate the antidepressant-like effects of magnesium and zinc ions [41]. In the present study, Se (0.25 mg/kg), when co-administered with DPCPX (1 mg/kg) and IST (0.5 mg/kg), significantly enhanced the activity of the tested mice in the FST and in the TST. These animals were swimming/climbing or struggling for a longer time than their vehicle-treated counterparts. It should be mentioned that the selected doses of interacting agents were individually ineffective in both tests.

Data in the literature encourages the application of both despair behavioral tests for evaluating the antidepressant potential of a given new agent or a new combination of known substances because the FST and the TST are not equally sensitive to drugs. For example, the effects of non-typical drugs with antidepressant potential are better pronounced in the FST [37,44], whereas it is believed that the mouse FST is not the best option to detect SSRI activity [36,37]. It should be remembered that the FST and the TST are based on the same principle, but they differ in relation to the neurological mechanisms that underlie the observed antidepressant activity.

Since it is widely known that effects observed in the FST and the TST may be positively falsified by a drug-induced hyperactivity, we checked whether the tested doses of DPCPX, IST, and Se as well as their respective combinations affected the spontaneous locomotor activity of mice. None of the tested substances or their co-administration significantly changed rodents’ performances when they were put into cages of the monitoring device. This was in line with our previous studies on DPCPX [41,43] and IST [41].

As no spontaneous hyperlocomotion was detected, we can suggest that the increased mobility of the animals that received both Se and DPCPX or IST in the FST and in the TST was a result of the synergistic antidepressant-like effect of the tested agents. Since the trend was the same in the FST and in the TST, the observed animal behavior could not have been a pure coincidence or an effect induced by environmental factors.

As we observed before, selective A1 and A2A receptor antagonists may interact differently with other substances with respect to their antidepressant potential. When a selective A2A receptor antagonist, DMPX, increased the activity of agomelatine and tianeptine in the FST and in the TST, the co-administration of DPCPX with these antidepressant drugs did not influence their activity [42]. However, both DPCPX and DMPX potentiated the effects of moclobemide, venlafaxine, and bupropion in the FST [40]. In the present study, both applied adenosine receptor antagonists interplayed with Se in the same manner.

Casaril et al. [38] suggested that the antidepressant-like activity of selenium compounds may be due to their anti-inflammatory and antioxidant properties. In fact, earlier reports gave evidence that selenium is crucial for the appropriate functioning of the CNS and that it acts as a protective compound, particularly in the hippocampus, striatum, and prefrontal cortex [7,8,9,10,45,46]. Furthermore, selenium modulates the main neurotransmissions, such as dopaminergic [28,47], GABA-ergic [48,49,50], cholinergic [51], and glutamatergic [20] signallings, and it modifies the influx of calcium into neurons [11,12,52].

Though using male mice only may be perceived as a limitation of this study, we did it on purpose. Since we regarded the present experiments as the preliminary ones, we did not want to obtain results that could potentially be affected by the oestrous cycle and its variations. Certainly, our promising findings should be confirmed in female subjects, considering the fact that prevalence of major depression is even higher in women than in men [53].

*BDNF level*. BDNF is a key member of the neurotrophic family and plays an important role in stress-related depression. The neurotrophic hypothesis of depression posits that chronic stress is associated with a reduction in BDNF and the resulting atrophy of neurons in brain regions associated with this disease [54]. It plays an important role in the maintenance and survival of neurons, and it regulates neurogenesis in the brain [55]. Animal studies have shown that BDNF expression is dysregulated by stress [56]. The administration of corticosterone to animals has been shown to reduce the expression of BDNF in the brain [57]. In particular, stressors such as forced swimming significantly reduce the expression of BDNF in the hippocampus [58]. However, antidepressants have been shown to prevent the stress-induced reduction of BDNF and to restore the corticosterone-dependent decline in BDNF expression [59,60]. Studies have found that antidepressant treatment increases swimming time and increases the BDNF mRNA in an animal model [61]. Changes in the BDNF level in the serum of patients with depressive disorders mean that BDNF can be considered as a biomarker of this disease [62]. However, since the level of BDNF in the brain of depressed patients is impossible to measure and in vivo studies have shown that central and peripheral BDNF levels are related to each other, blood levels of BDNF are determined in patients [63]. Therefore, in our study, we assessed the concentration of this biomarker in the blood of mice. Our research revealed that neither DPCPX nor IST or Se administered alone induced statistically significant changes in BDNF levels in mice serum when compared to the NaCl-treated group. There were also no statistically significant changes in comparison to the NaCl-treated group in any of the groups receiving the combination of tested compounds. Only in the group that received DPCPX + Se was a significant decrease in the level of BDNF noticed when compared to the DPCPX-treated group. However, it should be noted that BDNF counteracts the adverse effects of stress-induced glucocorticoid signaling, and it has therefore been linked as a factor of resistance to chronic stress-induced psychopathology [64]. While the effects of chronic stress on BDNF levels are well understood, much less is known about effects of acute stress on BDNF levels [64]. The effect of acute stress on peripheral BDNF levels in humans is not fully elucidated, and the relationship between blood BDNF levels and cortisol in response to acute stress is still unexplored. By analyzing results of behavioral tests and by considering the above considerations, it can be assumed that the lack of expected changes in BDNF levels in mice may be related to the duration of the stressors. Studies have shown that the upregulation of the BDNF occurs after the long-term administration of antidepressants in line with the time course of the therapeutic effect of antidepressants [63,65,66].

*Gene Expression Analysis.* It is known that stress plays an important role in the development of depression. In turn, stress causes the excessive generation of ROS (reactive oxygen species), leads to mitochondrial failure, oxidative stress, and, consequently, neurodegeneration [67]. In depressive disorders related to stress factors, the rate of oxygen conversion to ROS may increase and then may result in severe metabolic dysfunction and oxidative damage to the lipids and enzymes of the cellular and subcellular membranes [68,69]. It has been proved that an important mechanism in depression is the modulation of the expression of genes related to oxidative stress [70,71]. Therefore, the aim of this study was also to evaluate the expression of selected genes related to oxidative stress in the cortex of mice. The FST is a widely accepted model of behavior similar to depression and physical stress research. Research has shown that stress in the form of forced swimming can activate free radical processes [72,73].

The 8-oxoguanine glycosylase1 (*OGG1*) gene plays a key role in DNA repair pathways because it encodes an enzyme responsible for the excision of 8-oxoguanine, which is a mutagenic byproduct of oxidative stress [74,75]. Methionine sulfoxide A reductase (MSRA) also plays an important role in repairing proteins damaged as a result of oxidation, which restores their biological activity [76]. MSRA reduces methionine sulfoxide (MetO) to methionine, the residues of which are particularly vulnerable to oxidation by ROS. Therefore, MSRA plays an important role in cellular metabolism as an antioxidant enzyme [77,78]. As our research showed, IST and DPCPX + Se significantly reduced the relative level of *Ogg1* mRNA compared to the NaCl-treated group. The co-administration of DPCPX with Se resulted in a significant decrease in *Ogg1* expression compared to both the DPCPX-treated and Se-treated groups of mice. In our study, IST alone significantly reduced the relative level of *Msra* mRNA compared to the NaCl-treated group. Research has shown that stress leads to the overexpression of DNA repair enzymes important for the maintenance of mitochondrial function arising from stress-related metabolic disorders [79]. This is most likely due to the need to defend cells against the over-generation of ROS. Studies have shown that the overexpression of *Msra* prolongs the life of mice and human T lymphocytes under oxidative stress [76,78]. Therefore, the reduction in the mRNA level of antioxidant defense enzymes observed in our research may suggest the effectiveness of the tested substances in preventing the formation of oxidative stress. On the other hand, it should be noted that lowering the level of antioxidant defense enzymes may be unfavorable in a stressful situation, when there is an increased generation of ROS and the formation of oxidative stress. Thus, in order to accurately interpret the effects of single and combination therapy with the tested substances, studies in the chronic stress model should be carried out. This will allow for the verification of the above hypothesis and the assessment of the effectiveness of the tested substances in chronic stress. Moreover, as revealed by the results of our study, a significant reduction in *Ogg1* expression compared to the control was noted in the groups of mice receiving IST alone and IST + Se, while DPCPX alone did not reduce the expression of this gene. As in the above considerations, on the one hand, it may indicate a more effective defense against excess ROS after combined therapy (stress leads to the overexpression of DNA repair enzymes); however, on the other hand, it may suggest a weakened antioxidant defense under stress conditions, especially considering that there were no changes when administering DPCPX alone. These results are difficult to interpret unequivocally; further studies in the chronic stress model would provide valuable information in this regard. Interestingly, in animals receiving IST + Se, a statistically significant increase in the level of *Ogg1* mRNA compared to the IST-treated group was observed. Similarly, the co-administration of IST with Se resulted in a significant increase in the *Msra* expression in comparison to the IST-treated group. However, it should be noted that the increase in gene expression with the concurrent treatment of IST and Se was only compared to IST alone; no changes were observed when compared to the control group. The lack of overexpression with respect to the antioxidant defense genes when compared to the control does not indicate the participation of combined Se and IST therapy in the over-generation of ROS but may only suggest a weaker defense effect when compared to the treatment of IST alone.

Factor 2 associated with nuclear erythroid factor 2 (NRF2) is the regulator of cellular resistance to metabolic changes caused by oxidative stress. It plays a regulatory role in maintaining the balance of antioxidant enzymes [80]. In the mechanism of cellular defense against excess ROS, it occurs through the activation of the signaling pathway of the anti-oxidant element of the Nrf2 factor. This pathway participates in the control of the expression of genes whose protein products are involved in the detoxification and elimination of reactive metabolites [81,82]. It has been observed that the overexpression of *NRF2* is an important defense element in neurodegenerative diseases [83], while a reduction in *NRF2* activation may, in turn, lead to the weakening of the antioxidant response [84]. Our studies showed that DPCPX alone significantly increased, where IST and Se decreased, the relative mRNA level of *Nrf2* when compared to the NaCl-treated group. Conversely, the co-administration of DPCPX with Se resulted in a significant reduction in *Nrf2* expression when compared to the group treated with NaCl and DPCPX. Additionally, in the case of the IST + Se group, there was a significant increase in the level of *Nrf2* mRNA compared to the group treated with IST and Se. Regarding the above considerations, it can be assumed that the treatment of DPCPX alone is very beneficial in this context; however, single treatment with the remaining substances and the combined treatments of Se with DPCPX and Se with IST may weaken the preventive effect against excess ROS. It should be emphasized that NRF2 is a factor regulating the transcription of DNA oxidative damage repair genes [85], so the obtained results should be considered in relation to the results of changes in the expression of *Ogg1* and *Msra* genes. In the case of treatment with DPCPX alone, no changes in their mRNA levels were observed. Thus, the recorded *Nrf2* overexpression may indicate a protective effect by first activating the regulatory agent. In the case of treatment with IST alone or Se with DPCPX, where a decrease in the expression of antioxidant defense genes was also observed, we can presume that the interrelationships between the changes in the levels of the examined factors occur on other levels. It should be noted, however, that the reduction in the *Nrf2* mRNA level was also observed in the remaining groups (Se with IST), where no changes in the expression of *Ogg1* and *Msra* genes were noted compared to the control. When comprehensively analyzing the obtained data, they appear surprising and difficult to explain unequivocally; more research is needed in this area.

In our work, we also performed the expression of selected genes that play a key role in the pathomechanism of depression but may also play a role in the mechanisms of action of DPCPX, IST, and Se. SLC6A15 is a neutral amino acid transporter and is mainly expressed in neurons. It is presented as a candidate gene for major depression and stress susceptibility. SLC6A15 plays a role in modulating emotional behavior, possibly mediated by its effects on glutamatergic neurotransmission [56]. It has been shown that the reduced expression of *SLC6A15* in the brain can increase susceptibility to stress by altering the neural integrity of the excitatory neurotransmission [86]. Our study showed a very significant increase in the expression of *Slc6a15* in the brains of mice subjected to combined Se and DPCPX therapy, both compared to the control group and to groups receiving the tested drugs alone. Interestingly, DPCPX and Se alone significantly reduced the relative level of *Slc6a15* mRNA compared to the NaCl-treated group. It is possible that during the joint administration of these substances, changes in the neurotransmission of glutamate occur, e.g., by modulating the activity of other ionotropic and metabotropic glutamate receptors, which is not observed with single therapies. It is also possible that the increased expression of this gene is due to an effect on the regulation of the hypothalamic–pituitary–adrenal (HPA) axis. Regardless of the above, such a significant increase in the expression of the studied gene appears to be positive in the context of the potential use of the combined antidepressant therapy of Se and DPCPX. It may also indicate the mechanism of this action related to the modulation of the *Slc6a15* gene, the functional role of which in relation to the development of depressive disorders and the mechanisms of modulation through treatment is still unclear.

Catechol-O-methyltransferase (COMT) is closely related to depression [87]. The COMT enzyme is expressed in the brain, and it breaks down dopamine as well as other catecholamines and sex steroids. Animal and human studies have shown that altered levels of dopamine neurotransmission contribute to depression-like behaviors and that they influence depressive symptoms [88,89]. The involvement of COMT in monoamine metabolic pathways indicates the pleiotropic effect of this gene on the susceptibility to psychiatric disorders and symptoms [90]. Substances with antidepressant activity, by increasing the transmission of catecholamines, may influence the activity of enzymes involved in their metabolism and thus may regulate levels of COMT. In our study, we observed that IST and Se alone as well as IST administered with Se significantly increased the relative level of *Comt* mRNA compared to the control group. The explanation for these changes may be the hypothesis that, during treatment, the level of catecholamines in the brain increases, and then the activity of COMT, as an enzyme responsible for the breakdown of catecholamines, is greater. However, the increase in COMT activity, which may lead to an increase in the metabolism of neurotransmitters, is a disadvantageous phenomenon. In contrast, the co-administration of DPCPX with Se resulted in a very significant decrease in *Comt* expression when compared to the group treated with NaCl, DPCPX, and Se. When analyzing the obtained results in the context of favorable changes in the mRNA level of *Slc6a15* during combined therapy with Se and DPCPX, the reduction in the activity of enzyme-metabolizing neurotransmitters is very beneficial and it may be a key mechanism of antidepressant action.

We also disclosed that Se administered alone and with IST significantly increased the relative level of *Adora1* mRNA. *ADORA1* is a gene that modulates the release of neurotransmitters and encodes the adenosine A1 receptor, which is present in large amounts in the hippocampus and the cerebral cortex [91]. An endogenous agonist of the A1 receptor is adenosine, which, by acting on A1, inhibits the release of stimulant neurotransmitters [92]. The increase in *Adora1* expression is related to the increase in its activity at A1 receptors and is involved in the regulation of neurotransmitter release. It is known that adenosine receptors play a large role in pathogenesis as well as in the treatment of depressive disorders. Studies in mice suggest that increased A1 expression may have an antidepressant effect that is directly related to the function of astrocytes [93,94]. It has also been shown in a model of transgenic mice with the conditioned upregulation of brain A1 receptors that increasing A1 expression induces resistance to depressive behavior in behavioral tests, with it also exerting an antidepressant effect in the chronic stress model [95]. As in the case of *Comt*, the observed increase in the expression of *Adora1* may result from an increase in the concentration of neurotransmitters; increased neurotransmission may lead to stimulation of the A1 receptor and indicate the beneficial effects of the action. Conversely, the co-administration of DPCPX with Se resulted in a significant decrease in *Adora1* expression. Taking into account the above considerations and favorable changes in the expression of other genes, these results are surprising. It is worth noting that the non-selective inhibition of adenosine receptors in the CNS induces antidepressant-like behavior in animals and that there is an association between adenosine modulation and dopaminergic and glutaminergic transduction [96]. It is possible that the reason for the observed differences are the complex mechanisms of interactions between the adenosine and glutamatergic systems, which may be related to the effects on many types of receptors. It should also be emphasized that both the stress response and the response to treatment do not always involve the coordinated regulation of genes, the levels of which are regulated by many independent factors.

## 4. Materials and Methods

### 4.1. Animals

Adult naïve male Albino Swiss mice (*n* = 230) purchased from a licensed breeder (Experimental Medicine Center (OMD), Medical University of Lublin) were housed in groups of 10 in environmentally controlled rooms (temperature 22 ± 1 °C, relative humidity 45–55%) with a 12 h light/dark cycle with lights on at 8:00 a.m. Throughout the research, the animals were given free access to standard rodent chow and water. All procedures were carried out between 9 a.m. and 3 p.m., in accordance with the European Committee Directive for Care and Use of Laboratory Animals (2010/63/EU) and were accepted by the Local Ethics Committee.

### 4.2. Drug Administration

Sodium selenite (Se), DPCPX (8-cyclopentyl-1,3-dipropylxanthine), istradefylline (IST, (E)-8-(3,4-dimethoxystyryl)-1,3-diethyl-7-methylxanthine), and imipramine hydrochloride (IMI) were purchased from Sigma-Aldrich (Poznań, Polska). Se was dissolved in 0.9% NaCl and administered intraperitioneally (i.p.) 30 min prior behavioral testing at doses of 0.125, 0.25, 0.5, and 1.0 mg/kg, which refers to 0.056, 0.1125, 0.225, and 0.45 mg/kg of pure selenium, respectively. DPCPX and IST were suspended in 1% solution of Tween 80 (POCH, Gliwice, Polska) in saline (0.9% NaCl). DPCPX was injected i.p at a dose of 1 mg/kg, while IST was administered per os (p.o.) at a dose of 0.5 mg/kg 60 min before the experiment. IMI (30 mg/kg), used as a reference drug (a positive control group) in Se dose-effect studies, was dissolved in 0.9% NaCl and injected i.p. 60 min before behavioral studies. The control group received 0.9% NaCl at the respective time prior to the experiment. All solutions/suspensions were prepared immediately before behavioral tests and administered in a volume of 0.01 mL/g body weight. Each experimental group consisted of 10 mice, randomly assigned prior to administration of a tested substance.

#### Administration Schedule

The doses and administration schedule for the tested substances were selected based on the literature data and our previous research [29,40,41,42,43].

*Experiment 1—Sodium selenite dose-effect studies*
  I. NaCl (control group)
  II. Se 0.125 mg/kg
  III. Se 0.25 mg/kg
  IV. Se 0.5 mg/kg
  V. Se 1.0 mg/kg
  VI. IMI 30 mg/kg (positive control group)

*Experiment 2—Influence of selective adenosine A1 and A2A receptor antagonists on the antidepressant-like activity of sodium selenite*
  I. NaCl + NaCl (control group)
  II. Se 0.25 mg/kg + NaCl
  III. DPCPX 1 mg/kg + NaCl
  IV. DPCPX 1 mg/kg + Se 0.25 mg/kg
  V. IST 0.5 mg/kg + NaCl
  VI. IST 0.5 mg/kg + Se 0.25 mg/kg

### 4.3. Behavioral Tests

#### 4.3.1. Forced Swim Test (FST)

The FST was carried out according to the procedure described in detail previously [40,41,97]. The test was video recorded, and the total duration of immobility was measured between the 2nd and the 6th min of the test by two blind observers. A given mouse was regarded as immobile when it made only movements necessary to float passively in the water.

Results of the FST were expressed as the mean of immobility time of animals (s) ± standard error of the mean (SEM) for each experimental group.

#### 4.3.2. Tail Suspension Test (TST)

The TST was carried out according to the procedure described in detail previously [40,98]. The TST was video recorded, and the total duration of immobility was measured between the 2nd and the 6th min of the test by two blind observers. An animal was regarded as immobile when it made only movements necessary to breathe.

Results of the TST were expressed as the mean of immobility time of animals (s) ± SEM for each experimental group.

#### 4.3.3. Spontaneous Locomotor Activity Test

The measurement of spontaneous locomotor activity was carried out in an actimeter, the Opto-Varimex-4 Auto-Track (Columbus Instruments, Columbus, OH, USA), according to the procedure described in detail previously [40,41]. The total distance travelled by mice was measured (cm) automatically during the last 4 min of the 6-min testing period, which corresponded with the time intervals estimated in the FST and that TST.

Results of the spontaneous locomotor activity were expressed as the mean of distance travelled by animals (cm) ± SEM for each experimental group.

### 4.4. Biochemical Analysis

#### 4.4.1. Collection of Blood and Brain

Immediately after behavioral testing, mice were decapitated in order to collect the blood and the prefrontal cortex for the analysis of BDNF levels and the expression of selected genes.

The blood was collected in Eppendorf tubes, and it was left at a temperature of 20–25 °C for 30 min. The clotted blood was centrifuged at 5000 rpm for 10 min. Then, serum was transferred by an automatic pipette to new Eppendorf tubes, and it was frozen at −80 °C until the BDNF level was analyzed. The brains of mice were carefully removed and rinsed with ice-cold saline (2 °C) in order to remove blood. Next, the prefrontal cortex was isolated and stored in a freezer at −80 °C in Eppendorf tubes until the expression of selected gens was analyzed.

#### 4.4.2. Evaluation of the BDNF Level

Determination of the BDNF level in mice serum was carried out according to the method described in detail previously [41]. A diagnostic kit for an enzyme-linked immunosorbent assay (ELISA) in murine serum (Enzyme-linked Immunosorbent Assay Kit For BDNF, Cloud-Clone Corp., Katy, TX, USA) was used in accordance with the producent’s instructions.

#### 4.4.3. Evaluation of the Relative mRNA Level of Selected Gens

Determination of the *Ogg1*, *Msra*, *Nfe2l2*, *Adora1*, *Comt*, and *Slc6a15* relative gen expression was measured in the murine prefrontal cortex in accordance with the method described in detail previously [41].

Firstly, to isolate the total RNA from the murine prefrontal cortex, the TRIzol Reagent (Invitrogen, Carlsbad, CA, USA) was used according to the manufacturer’s instructions. The level and purity of RNA were determined with a NanoDrop Maestro Nano spectrophotometer (Maestrogen, Hsinchu, Taiwan). RNA of the highest purity (A260/280 ratio ranged between 1.8 and 2.0) was used for subsequent determinations.

The second step of gen expression studies was cDNA synthesis, which was performed using a high-capacity cDNA reverse transcription kit (Applied Biosystems, Foster City, CA, USA) according to the producent’s instructions. The reaction took place under the following conditions: 10 min at 25 °C, 120 min at 37 °C, and 5 min at 85 °C to end the process. cDNA obtained in this procedure was kept at −20 °C until further analysis.

The final stage was the performance of real-time PCR. The procedure was carried out using the 7500 Fast Real–Time PCR System (Applied Biosystems, Foster City, CA, USA) and Fast Probe qPCR Master Mix (EURx, Gdańsk, Poland) with plus ROX Solution (EURx, Gdańsk, Poland) according to the producent’s instructions. The ΔΔCt method was chosen, as were *Hprt* and *Tbp* as endogenous controls. The reaction took place under the following conditions: 3 min at 95 °C × 1 cycle, 10 s at 95 °C, and 30 s at 60 °C × 40 cycles.

To eliminate any outlier outcomes before ΔΔCt estimations and to establish the fold change in mRNA levels, the data quality screen based on amplification, i.e., T_m_ and C_t_ values, was carried out. The results of real-time PCR were shown as RQ value (RQ = 2 − ΔΔCt).

### 4.5. Statistical Analysis

The statistical analysis of the results obtained in the FST, TST, and locomotor activity assessment following Se administration was carried out using one-way ANOVA with Dunnett’s post-hoc test. Also, outcomes of the evaluation of BDNF levels in mice serum were analyzed using the same statistical test. Additionally, after co-treatment with Se and the selective adenosine antagonists of A1 or A2A receptors, a two-way ANOVA followed by the Bonferroni’s post hoc test was used to analyze results from behavioral tests and from analyses of gene expression. Differences between groups were considered statistically significant when *p* ≤ 0.05.

## 5. Conclusions

Taken together, the results of our research confirmed that sodium selenite administered at a single dose may exert an antidepressant-like effect in behavioral tests in mice, and that this effect is dose-dependent. Furthermore, sodium selenite is able to interact with selective antagonists of adenosine A1 and A2A receptors. A consequence of this interaction is the enhancement of activity of mice in the FST and the TST, and this effect is not due to the hyperlocomotion of the experimental animals. The lack of changes in BDNF levels in mice, noted in our research, may be related to the short duration of the stress factor. The changes in the mRNA level of antioxidant defense enzymes observed in our research may, on the one hand, suggest the effectiveness of ISTRA and the combined DPCPX + Se therapy in preventing oxidative stress. On the other hand, they may indicate a weakening of the antioxidant defense by lowering the level of defense enzymes. In order to explain the above assumptions, studies in the chronic stress model are necessary. In turn, changes in the mRNA level of *Slc6a15* and in gene-encoding, enzyme-metabolizing neurotransmitters, during the combined therapy of Se with DPCPX, indicate a beneficial effect and may be a key mechanism of an antidepressant action. The combination of sodium selenite with substances attenuating adenosine neurotransmission may become a new therapeutic strategy for patients with depressive disorders. Certainly, our promising findings should be confirmed in further studies.

## Figures and Tables

**Table 1:** Effect of sodium selenite on mice spontaneous locomotor activity.

Treatment [mg/kg]	Distance [cm] between the 2nd and the 6th min
NaCl 0.9%	987.3 ± 60.71
Se 0.125	975.0 ± 74.68
Se 0.25	986.1 ± 31.71
Se 0.5	1036 ± 61.33
Se 1.0	1137 ± 46.61
IMI 30	793.3 ± 41.62

Sodium selenite (Se) and NaCl 0.9% were administered i.p. 30 min, while IMI was given 60 min prior behavioral testing. The travelled distance was recorded between the 2nd and the 6th min of the test. Each experimental group consisted of 10 animals. Data are presented as the means ± SEM (one-way ANOVA followed by Dunnett’s post-hoc test).

**Table 2:** Effect of co-treatment on mice spontaneous locomotor activity.

Treatment [mg/kg]	Distance [cm] between the 2nd and the 6th min
NaCl 0.9% + NaCl 0.9%	1196.5 ± 58.3
Se 0.25 + NaCl 0.9%	1051.7 ± 43.8
DPCPX 1.0 + NaCl 0.9%	1111.8 ± 51.7
DPCPX 1.0 + Se 0.25	922.20 ± 79.9
IST 0.5 + NaCl 0.9%	1093.2 ± 54.7
IST 0.5 + Se0.25	1109.6 ± 65.8

DPCPX, sodium selenite (Se), and NaCl 0.9% were administered i.p. 30 min, while IST was administered p.o. 60 min prior locomotor activity test. The travelled distance was recorded between the 2nd and the 6th min of the test. Each experimental group consisted of 10 animals. Data are presented as the means ± SEM (two-way ANOVA followed by Bonferoni’s post-hoc test).

## Data Availability

The data presented in this study are available in article.
